# Mono‐ and Bivalent Poly(*iso*‐butylene)‐Alanines for Drug‐Delivery of Nimodipine and Triamcinolone Acetonide

**DOI:** 10.1002/marc.202500321

**Published:** 2025-09-18

**Authors:** Philipp S. Hilgeroth, Julius F. Butter, Wolfgang H. Binder

**Affiliations:** ^1^ Design of 3D‐Printable Polymers Based on Regional Resources Just Transition Center Martin Luther University Halle‐Wittenberg Halle Germany; ^2^ Macromolecular Chemistry Division of Technical and Macromolecular Chemistry Faculty of Natural Sciences II (Chemistry, Physics, Mathematics) Institute of Chemistry Martin Luther University Halle‐Wittenberg Halle Germany

**Keywords:** living carbocationic polymerization, nimodipine, poly‐*iso*‐butylene, secondary structures, triamcinolone acetonide

## Abstract

Poly‐*iso*‐butylene (PIB) is a well known biocompatible polymer with application as a solid drug delivery system. However its applications are limited by its soft mechanical properties, necessitating the introduction of assembling structural elements to modify its microstructure by reinforcing elements to achieve structural stability. We here investigate the impact of embedded oligo‐alanine‐units (Ala) to induce beta‐sheets and the release of drugs from a so solidified PIB, prepared by living carbocationic polymerization (LCCP). Mono‐ (PIB‐Ala_2_‐Ac, 12,2 kDa) and bivalent PIB‐polymers (PIB‐(Ala_2_‐Ac)_2_, 16,7 kDa) with attached oligo(alanines) as endgroups were synthesized and induce beta‐sheets to adjust the mechanical strength of these biomaterials. Mechanical and thermal properties are investigated using temperature dependent IR spectroscopy and melt‐rheology rheology, with circular dichroism (CD) proving the formation of stable beta‐sheets for PIB‐(Ala_2_‐Ac)_2_ inside the solid polymer. Two drugs, nimodipine and triamcinolone acetonide, are incorporated into different PIB matrices to study the influence of drug—secondary structure interactions. The polymer/drug formulations display a stable release of the drugs over a period of 42 days into a PBS buffer, thus presenting an attractive material for long‐term targeted release with adjustable mechanical properties.

## Graphical abstract

Mono‐ and bivalent‐, alanine‐modified poly‐*iso*‐butylenes, folding into beta‐sheets, were prepared by living carbocationic polymerization and subsequent endgroup modification. These PIB‐conjugates are useful as solid polymers for the drug release of triamcinolone and nimodipine.

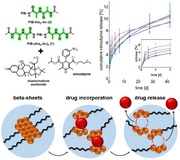

## 1 Introduction

Poly‐*iso*‐butylene (PIB) and its co‐polymers have gained attention due to their excellent biocompatibility [1], explored for various biomedical applications [2, 3, 4]. The TAXUS drug‐eluting stent displays excellent vascular compatibility [5] and biostability [6] proven over a period of up to 360 days [7], so allowing long term implantation. In recent years PIB‐segments have been further introduced into polyurethane films, improving oxidative stability [8, 9] and showing excellent hydrolytic resistance, along with minimal swelling in water [10]. These films show an only minor decrease in performance [11], showing potential as candidates for the fabrication of fully synthetic prosthetic heart valves [12]. To improve mechanical stabilities and drug‐elution properties co‐polymers of PIB, poly(styrene‐*b*‐*iso*‐butylene‐*b*‐styrene) (SIBS), are applied in the TAXUS stent, delivering paclitaxel directly to arterial walls thereby preventing restenosis [13]. Structural stability of the normally too soft PIB is reached by microphase separation of the two polymer segments, so providing optimal long term drug release conditions after an initial burst release [14]. Besides its use as a carrier for paclitaxel [15] SIBS is also applied for the release of tetracycline, proving the distribution and interaction of this drug in specific SIBS microphases in dependence of the poly‐*iso*‐butylene (PIB)‐to‐polystyrene (PS) ratio [16]. Dexamethasone was embedded in different formulations into high molecular weight SIBS polymers, probing the influence of sample preparation via electro‐spraying [17]. Zafirlukast was introduced to a mixture of polyethylene glycol (PEG) and a hyperbranched SIBS co‐polymers with *p*‐methylstyrene for the preparation of films using electrospinning, thus creating self‐standing fiber mats [18], which display release kinetics of over 90% over a period of 21 days. We previously have studied the 3D printability of low molecular weight SIBS and triamcinolone acetonide formulations, monitoring impact of elevated temperatures and high forces onto the drug release kinetics [19]. Transdermal applications were also explored using PIB as pressure sensitive adhesives (PSAs) for the successful and controlled release of seral drugs like lidocaine [20], tranylcypromine [21], and olanzapine [22].

Controlling the mechanical properties of PIB therefore is one of the main challenges to enable its wider use in medical applications in a 3D‐printable format. Therefore, microphase segregation, creating soft and hard segments, can be used to reach an optimal balance between drug incorporation and mechanical stability. Croiser et al. [23] studied a material that embeds specific nanostructures into a PIB matrix by use of oligo peptides, that act as reinforcing elements inside the solid PIB. The covalently attached oligo‐alanines showed a significant improvement of mechanical strength of low molecular weight PIB (2000 g mol^−1^) due to the formation of secondary structures such as beta‐sheets inside the solid PIB‐matrix.

**SCHEME 1:**
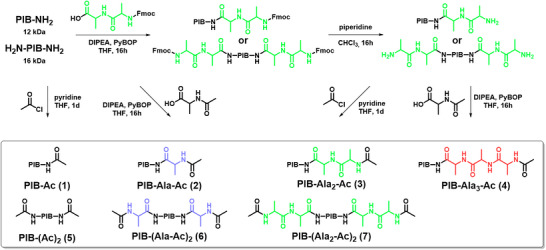
Synthetic pathway for modification of mono‐ and bivalent, for the attachment of oligo amino acids onto PIB‐amines (mono‐/bivalent).

We here focus on the use of an alanine‐modified PIB (see Scheme 1) embedding two drugs, where the release from solid implanted polymers is important: nimodipine (NMP) is a well‐known calcium channel blocker and is used to prevent vasospasm after subarachnoid hemorrhage; triamcinolone acetonide (TA) is an antiinflammatory drug. The embedding of the oligoalanine units follows a novel synthetic pathway based on a de novo synthesis of an amine‐modified PIB via living carbocationic polymerization in combination with solid phase synthesis of amino‐acids, currently not achieved by previous authors. The folding elements, Ala, were further attached by peptide synthesis to an amino‐terminal‐PIBto reach the precisely structured PIBs, then organized into beta‐sheets. We use those polymers for the delivery of drugs currently used in polymer formulations with poly‐(lactic‐*co*‐glycolic acid) (PLGA), poly‐caprolactone (PCL) or poly‐ethylene glycol (PEG), either in the form of micelles [24], micro emulsions [25] or nanocarriers [26] for targeted delivery to the eye [27] and the brain. Triamcinolone acetonide (TA) is a synthetic corticosteroid that is used as an anti‐inflammatory drug in both systemic or topical applications. It is commonly formulated with polymers such as chitosan, PEG, PCL, PLGA or poly butylene succinate (PBS) for extrusion via electro spinning [28, 29] or in micelles [30], liposomes [31] and nanoparticles [32] used for ocular treatments.

In this article we explore the potential of nanophase segregation inside the PIB‐polymer to enable a controlled release of these two different drugs, nimodipine and triamcinolone acetonide, while retaining good mechanical properties and study the inflence of both drugs on the structural organization. The designed either mono‐ or bivalent PIB, equipped with alanine‐units at the chain ends, gain structural stability and a tunable mechanical performance. We here report on the synthesis, and investigations on the secondary structure while monitoring stability and effects on the release kinetics of the two incorporated drugs NMP or TA.

## 2 Synthesis

Mono‐ and bivalent poly‐*iso*‐butylenes (PIBs) were synthesized via living carbocationic polymerization (LCCP) using 2‐chloro‐2,4,4‐trimethylpentane (TMPCl) or 1‐(tert‐butyl)‐3,5‐bis(2‐methoxypropan‐2‐yl)benzene as initiators (Scheme 1; Figure S1) to polymerize *iso*‐butylene as previously reported [33]. (3‐Bromopropoxy)benzene was used as a direct quencher, introducing a bromide end group with quantitative control, as indicated by NMR‐spectroscopy. The synthesized polymers (Table 1; Table S1) from both initiators yielded molecular weights from 12 kDa (monovalent) to 16 kDa (bivalent) with good PDIs (1.2–1.4) (Figure S5) and a high endgroup‐fidelity. The terminal bromide was then further modified using potassium phthalimide and hydrazine, to yield a terminal amine functionalization (Scheme S1) for a subsequent attachment of the alanine‐units [34].

**TABLE 1:** PDIs and molecular weights (GPC and NMR) from the synthesized polymer (PIB‐Br) and the final products after terminal functionalization.

Sample	Type	M_n_(GPC) [Da]	M_n_(NMR) [Da]	PDI
PIB‐Br	mono	12 700	15 400	1.39
PIB‐Ac **(1)**	mono	12 300	15 500	1.39
PIB‐Ala‐Ac **(2)**	mono	12 200	15 900	1.41
PIB‐Ala_2_‐Ac **(3)**	mono	12 400	15 400	1,41
PIB‐Ala_3_‐Ac **(4)**	mono	12 800	16 000	1,38
PIB‐(Br)_2_	bi	15500	15 900	1.21
PIB‐(Ac)_2_ **(5)**	bi	15 500	16 300	1.25
PIB‐(Ala‐Ac)_2_ **(6)**	bi	15 500	16 500	1.25
PIB‐(Ala_2_‐Ac)_2_ **(7)**	bi	16 700	16 600	1.21

N‐acetyl‐alanine‐OH was synthesized from alanine via coupling with acetyl chloride. Fmoc‐(Ala)_2_‐OH was then used for the following reaction steps in a sequential addition chemistry using standard‐peptide coupling (Scheme 1) with DIPEA and ByBOP in THF, followed by removal of the protection group. This resulted in either a mono‐ or a bis‐alanine modified PIB displaying the respective terminal amine‐groups. The amino terminated PIBs were further converted, using acetyl chloride or N‐acetyl‐alanine‐OH, yielding the desired polymers (Table 1) with the desired endgroup fidelity (Figures S1–S5).

## 3 Secondary Structure Analysis

**FIGURE 1:**
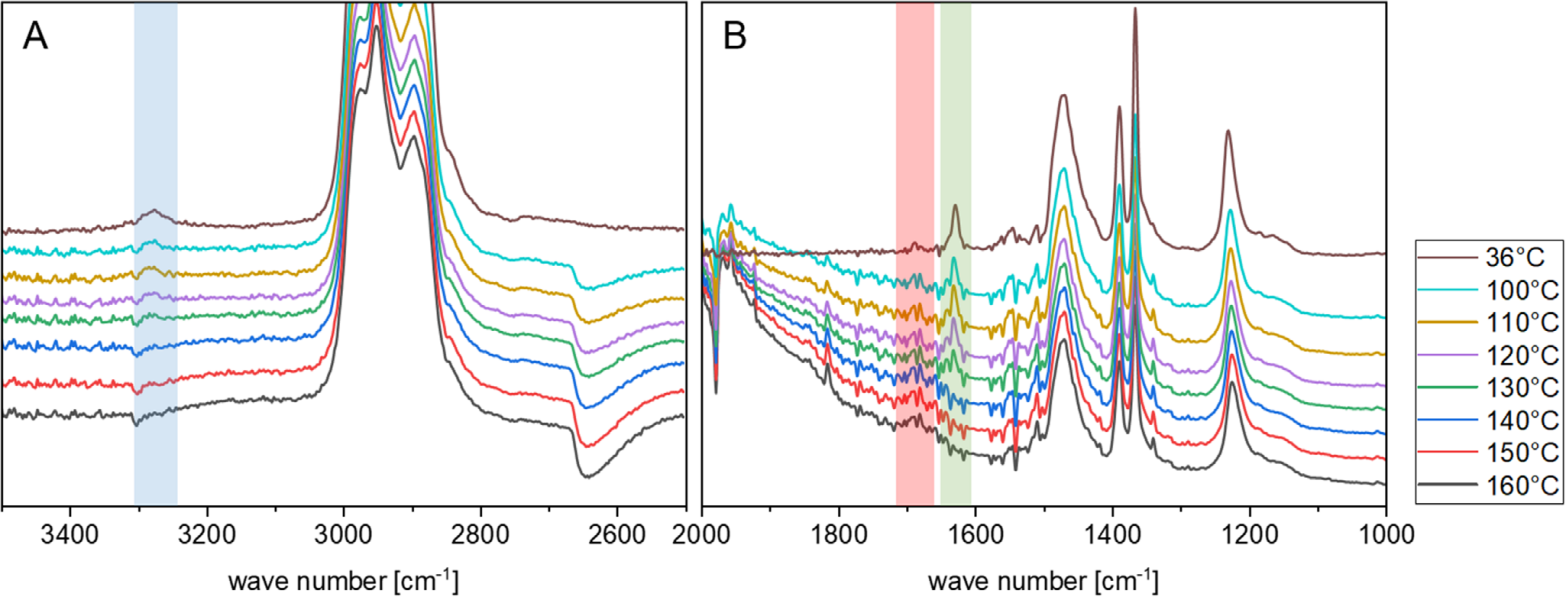
Temperature dependent IR measurements of polymer **(7)** with highlighted signals at 3275 cm^−1^ (blue), 1680 cm^−1^ (red), and 1630 cm^−1^ (green) representing ν(N‐H), free ν(C═O) and associated ν(C═O) respectively.

Based on previous investigations, the formation of beta‐sheets by oligo‐alanines attached to low molecular weight poly‐*iso*‐butylene chains (2000 Da) can be expected [23], and was studied using atomic force microscopy (AFM), X‐ray diffraction (XRD) and melt‐rheology, expecting the formation of beta‐sheets within the here projected solid polymer. To confirm the (temperature‐dependent) formation of beta‐sheets we conducted temperature dependent IR spectroscopy for PIB‐(Ala_2_‐Ac)_2_ **(7)** and all other polymers (Figure S7). The bands of associated N‐H are displayed at 37°C in the range of 3275 cm^−1^ (Figure 1A), indicating the formation of hydrogen bonds in the form of beta‐sheets. At elevated temperatures this band decreases and completely disappeared at temperatures above 150°C, similar for the C═O double bond bands (Figure 1B). The initial band of the associated C═O double bonds at 1630 cm^−1^ is shifting to 1680 cm^−1^ as the temperature increases, also indicating the thermal decay of some of the secondary structure. This behavior was further probed using DSC (Figure S8), where a transition is observed (150°C) for those sample containing larger fractions of Ala, indicative of the transition of the secondary structure, similar to structural changes in other polymers bearing oligomeric Ala‐endgroups [23]. Similar transitions have been previously  observed for glutamate‐bearing polymers, even though at far lower temperatures [35], and for aspartate‐modified polymers [36]. Notably, the addition of the terminal alanine units is not changing the glass transition temperature (*T*_g_) of the PIB‐polymer, which remains at almost unchanged at −60°C.

In order to confirm the hypothesis that beta‐sheets are the secondary structure element, solid state CD spectroscopy was conducted using an Ulbricht sphere for polymers the PIB‐Ala_2_‐Ac (**3**) and PIB‐(Ala_2_‐Ac)_2_ (**7**) (Figure S9). The measurement indicated the presence of beta‐sheets by an absorption maximum at 195 nm and an absorption minimum at 217 nm, which is the reported and expected pattern, in contrast to the main signals of alpha helices at 208 and 222 nm, further proving the formation of the predicted secondary structure in our system [37]. Notably both polymers, the mono‐ and bivalent polymers (**3**) and (**7**), are able to form beta‐sheets in the solid state, in addition to their 50:50‐mixture (Figure S9), which has been prepared by solvent‐based mixing and subsequent solvent removal.

**FIGURE 2:**
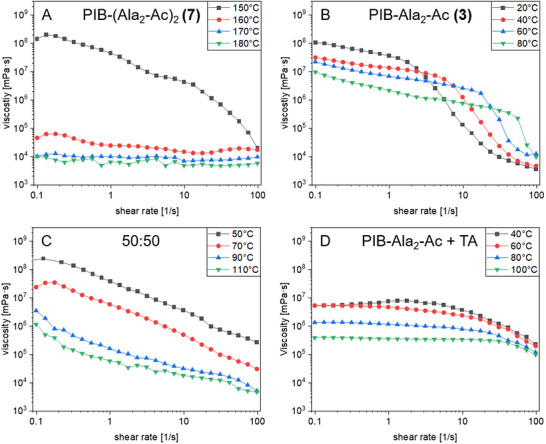
Temperature dependent rheological viscosity measurement of **(7)** (A), **(3)** (B), a 50:50 mixture of **(3)** and **(7)** (C), and **(3)** after incorporation of 5 wt.% TA (D).

Melt rheology was further performed to investigate the mechanical properties, e.g. melt flow behavior and shear viscosity, and check the form‐stability over time at different temperatures. We expect the formation of transient networks by the formed beta‐sheets, with a lower network density in the mixtures and the absence of network formation in the monovalent PIB‐Ala_2_‐Ac **(3)**. This will influence the mechanical properties and therefore further impact the drug release behavior. All measured polymer samples showed a significant increase in viscosity when compared to native PIB with a high viscosity (>10^5^ Pas) at room temperature (Figure S10). Particularly polymer **(7)** displays excellent mechanical stability up to 150°C (Figure 2A). In line with the IR measurements the viscosity is decreasing at 160°C, presumably caused by the deconstruction of the beta‐sheets. When compared to virgin monovalent PIB **(3) (**Figure 2B), a mixture (50:50) of **(3)** and **(7)** (Figure 2C) exhibits improved viscosity compared to virgin monovalent PIB‐Ala_2_‐Ac **(3)**.

## 4 Drug Incorporation

The incorporation of both drugs, triamcinolone acetonide (TA) and nimodipine (NMP), was achieved using solution blending using those two polymers (**3**) and (**7**) and their mixtures, which were rheologically found to be attractive for further use. Both, polymers and the respective drug were dissolved in THF and the solvent was subsequently evaporated, dried under reduced pressure at 37°C for a minimum of 24 hours to ensure its complete removal. The prepared formulations are described in (Table 2) .

**TABLE 2:** Polymer and drug contents of the prepared formulations with NMP and TA.

Sample	wt.% (3)	wt.% (7)	wt.% drug
Monovalent	95	0	5
75:25	71.25	23.75	5
50:50	47.5	47.5	5
25:75	23.75	71.25	5
Bivalent	0	95	5

In order to understand how the drugs are embedded into the polymers and to gain insight how the drugs are interacting with the hydrogen bonds of the beta‐sheets, rheology and grazing incidence wide angle X‐ray scattering (GIWAXS) were conducted. In rheology measurements it was observed that there are significant changes in the viscosity behavior upon incorporating the drugs. The virgin polymer samples exhibited a reduction in viscosity from > 10^5^ to < 10^2^ Pas at higher shear rates, indicative of the partial rupture of the hydrogen bonds within the beta‐sheets. After incorporation of TA, an almost linear behavior over the full temperature range is observed, with viscosities in the range of 4 × 10^3^ to 5 × 10^4^ Pas, up to very high shear rates (Figure 2D). CD spectroscopy was conducted for drug polymer formulations in the solid state, (Figure S9) further evidencing the disappearance of the characteristic beta‐sheet minima and the appearance of a new signal at 300 nm, now originating from the drug.

For an adequate understanding of the process of drug release, it is important to understand the mechanism underlying drug incorporation. If the drug is predominantly located on the surface, a high burst release would take place, thereby limiting the availability over extended periods of time. Also the formation of solid crystallites within the polymer matrix can result in the uncontrolled release of the drug, again resulting in bursts. Using GIWAXS of prepared thin films of the different formulations, significant differences were observed when comparing the drugs (Figure S11). NMP appears to be homogeneously dispersed in the formulations, while triamcinolone acetonide reflexes were observed in a 5 wt.% formulation, likely attributable to solid drug‐crystallites formed on the surface. In order to support these findings, atomic force microscopy (AFM) was conducted on various formulations as well as the virgin polymer samples (Figures S12–S17). Both drugs demonstrated agglomeration on the surface, with TA forming larger, crystal‐like structures, while NMP was primarily observed as amorphous residual material resulting from the evaporation of the solvent (Figures S12–S17).

## 5 Drug Release

**FIGURE 3:**
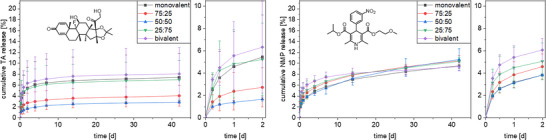
Drug release study of TA (left) and NMP (right) from different monovalent and bivalent bis‐alanine modified PIB mixtures, monitored using HPLC, over the time of six weeks.

The release of both drugs, TA and NMP, was studied in standard PBS buffer (pH 7.4) at 37°C from mixtures of monovalent PIB‐Ala_2_‐Ac **(3)** and bivalent PIB‐(Ala_2_‐Ac)_2_ **(7)** over a period of 42 days (Figure 3). All samples show a burst release after the first six hours. The daily release decreases over time, reaching a steady release rate after 4 days. Notably, both cumulative releases appear to be near‐zero order kinetic behavior after the first 4 days. Additionally no lag time was observed. The release rates were calculated from calibration curves (Figures S18, S19), with NMP demonstrating a daily release rates between 0.2 µg mL^−1^ for **(7)** and 0.6; µg mL^−1^ for **(3)**. In contrast, the TA formulations show uniform daily release rates of 0.07 ± 0.025 µg mL^−1^. Furthermore, these formulations experience a different burst release up to 50 µg mL^−1^ in the first day, while the daily release rates at later stages are consistent with expectations. This finding may originate from the crystallites formed on the surface during sample preparation, which were also observed in GIWAXS, as well as AFM.

## 6 Conclusion

Mono‐ and bivalent poly‐*iso*‐butylenes were synthesized via living carbocationic polymerization (LCCP) with molecular weights of 12 and 16 kDa respectively, carrying a distinct number of alanine‐units (Ala_1_, Ala_2_, Ala_3_) at their chain ends. The embedding of the oligoalanine units accomplished here, starting from the isobutylene‐monomer, follows a novel synthetic pathway, based on a de novo synthesis of an mono‐/bivalent amine‐modified PIB, currently not achieved by previous authors. Endgroup modification was achieved by successive attachment of alanine oligomers of varying lengths (n = 1–3) by subsequent peptide‐coupling to the amine‐terminated PIBs (mono/bi‐functional). The aggregation of the endgroups into beta‐sheets was investigated and proven using solid‐state CD spectroscopy. Two drugs (nimodipine or triamcinolone acetonide) were incorporated into the polymer matrix, which lead to changes in the CD spectra, indicative of an at least partial disassembly of the beta‐sheets, while retaining a transient network by the beta‐sheets, as demonstrated by melt‐rheology. Performed drug release studies showed a linear, near‐zero order release kinetic, thereby indicating a predominantly diffusion driven release, after an initial burst release, independent of the drug used, with varying release rates. The release system here presented offers potential for applications in slow release systems and can be adapted to suit different drugs and uses due to its adjustable release rates.

## Conflicts of Interest

The authors declare no conflicts of interest.

## Supporting information




**Supporting File**: marc70060‐sup‐0001‐SuppMat.docx.

## Data Availability

The data that support the findings of this study are available in the supplementary material of this article.
