# Supplementary material for: Mono‐ and Bivalent Poly(iso‐butylene)‐Alanines for Drug‐Delivery of Nimodipine and Triamcinolone Acetonide
Source: Macromol Rapid Commun. 2025 Sep 18;47(14):e00321. doi: 10.1002/marc.202500321 (PMC13384802; doi:10.1002/marc.202500321)
Supplement: Supplementary file 1 — Supporting File: marc70060‐sup‐0001‐SuppMat.docx. [file MARC-47-e00321-s001.docx]

Supporting Information

Mono- and Bivalent Poly(*iso*-butylene)-alanines as Conjugates for Drug-Delivery of Nimodipine and Triamcinolone Acetonide

Philipp S. Hilgeroth, Julius F. Butter, Wolfgang H. Binder*

Initiator-Synthesis

2,4,4-trimethylpentene (5 mL, 3.53 g, 36.0 mmol) was dissolved in dry DCM (20 mL) at 0°C. Gaseous HCl was formed from sodium chloride (50 g) and concentrated sulphuric acid (20 mL) and the solution was then bubbled with HCl for 5 hours, alternating between ten minutes of vigorous bubbling and 20 minutes of waiting. At the end of the reaction, the solvent was removed under vacuum at 0°C. Yield: 62%

^1^H NMR (400 MHz, CDCl_3_): δ 1.88 (H1, s, 2H), 1.67 (H2, s, 6H), 1.06 (H3, s, 9H).

5-(tert-butyl)isophthalic acid (0.02 mol) was dissolved in Methanol (175 mL) and H_2_SO_4_ (2 eq.) was added. The solution was stirred at 70°C for 2 days. The solvent was removed under reduced pressure, followed by column chromatography. Yield: 95%

^1^H NMR (400 MHz, CDCl_3_): δ 8.46 (s, 1H), 8.22 (s, 2H), 3.91 (s, 6H), 1.34 (s, 9H).

^13^C NMR (101 MHz, CDCl_3_): δ 166.57, 152.07, 130.85, 130.30, 127.97, 52.22, 34.92, 31.11.

Under inert gas conditions, magnesium chips (0.97 g, 40 mmol), 30 mL of dried diethyl ether and MeI (0.4 mL, 6.4 mmol) were added to the flask. Following a color change, additional MeI (2.6 mL, 42 mmol) was added. Afterwards, a solution of dimethyl-5-(tert-butyl)isophthalat (2 g, 8 mmol) and diethyl ether (35 mL) was added slowly over a period of 1 h. The reaction solution was stirred for 16 h. The reaction solution was then quenched in ice and NH_4_Cl. The product was then extracted thrice with diethyl ether, and dried over MgSO_4_. Yield: 77%

^1^H NMR (400 MHz, CDCl_3_): δ 7.42 (s, 3H), 1.71 (s, 2H), 1.60 (s, 12H), 1.34 (s, 9H).

^13^C NMR (101 MHz, CDCl_3_): δ 119.83, 117.64, 72.88, 31.87, 31.49.

2,2‘-(5-(tert-Butyl)-1,3-phenylene) bis-propan-2-ol (1.52 g, 4.5 mmol), was dissolved in 30 mL of MeOH. Subsequently, 4 μL of sulfuric acid (0.08 mmol) was added. The reaction mixture was stirred for 24 hours at 70°C, after which it was adjusted to a pH of 7 with NaHCO_3_. The solution was then extracted on twice with hexane, washed four times with water, and dried over Na_2_SO_4_. The solution was then purified by column chromatography. Yield: 15%.

^1^H NMR (400 MHz, CDCl_3_): δ 7.31 (d, J = 0.5 Hz, 2H,), 7.23 (t, J = 1.7 Hz, 1H), 3.07 (s, 6H), 1.53 (s, 12H), 1.33 (s, 9H).

^13^C NMR (101 MHz, CDCl_3_): δ 150.76, 145.19, 121.18, 120.35, 50.56, 34.84, 31.50, 28.13.

Polymerization

**Scheme S1.** Synthesis of PIB-NH_2_ from the two initiators.

A stock solution of DCM (1 mL), DTBP (59 μL, 0.32 mmol), DMA (10 μL, 0.22 mmol) and initiator (25 mg, 0.09 mmol) was prepared. The reaction flask, equipped with a mechanical stirrer, was then filled with hexane (13 mL) and DCM (8 mL). The stock solution was then added to the reaction flask and the flask was cooled to -80°C. Liquefied isobutylene (1.7 mL, 17.81 mmol) was then added to the reaction flask. TiCl_4_ (0.16 mL, 1.46 mmol) was then added to the reaction mixture and the reaction mixture was stirred for 15 min. The polymerization was quenched at -80°C for 5 h using (3-bromopropoxy)benzene. The resulting product was precipitated in methanol twice.

^1^H NMR (400 MHz, CDCl_3_): δ 7.27 (4H), 7,17 (s, 3H), 6.81 (d, J = 8.8 Hz, 4H), 4.08 (t, J = 5.8 Hz, 4H), 3.60 (t, J = 6.5 Hz, 4H), 2.31 (t, J = 6.2 Hz, 4H), 1.42 (s, 577H), 1.11 (s, 1727H).

Endgroup modification

N-Methyl-2-pyrrolidon (11 mL) was added to PIB-Br (0.21 mmol) dissolved in THF (22 mL). Potassium phthalimide (2.13 g, 11.56 mmol) was added and the mixture was stirred for 4 h at 70°C. The solution was diluted with hexane (50 mL), washed six times with water, and dried over MgSO_4_. The solvent was then removed under reduced pressure, and the product was dried under vacuum at 50°C. Yield: 90%

^1^H NMR (500 MHz, CDCl_3_): δ 7.84 (dd, J = 5.4, 3.0 Hz, 4H), 7.71 (dd, J = 5.4, 3.0 Hz, 4H), 7.22 (d, J = 8.8 Hz, 4H), 7.17 (s, 3H), 6.73 (d, J = 8.7 Hz, 4H), 4.02 (t, J = 6.1 Hz, 4H), 3.91 (t, J = 7.0 Hz, 4H), 2.18 (p, J = 6.5 Hz, 4H), 1.42 (s, 576H), 1.12 (s, 1732H).

PIB-Phth (2.5 g, 0.16 mmol) was dissolved in heptane (34 mL) and ethanol (34 mL) followed by the addition of Hydrazine hydrate (50%, 6 mL). The solution was stirred for 5 hours 110°C. The solution was then diluted with hexane (50 mL), washed six times with water, and dried over MgSO_4_. The polymer was precipitated in MeOH twice, then dried under vacuum for 20 hours. Yield: 74%

^1^H NMR (400 MHz, CDCl_3_): δ 7.16 (s, 3H), 6.81 (d, J = 8.9 Hz, 4H), 4.03 (t, J = 6.1 Hz, 4H), 2.91 (t, J = 6.8 Hz, 4H), 2.02 (p, J = 6.9 Hz, 4H), 1.41 (s, 540H), 1.11 (s, 1756H).

To solution of PIB-NH_2_ (100 mg, 0.00645 mmol) in THF (5 mL) was added to acetyl chloride (2 eq.) and pyridine (2.5 eq.). The mixture was stirred for of 24 hours. The polymer was washed twice with water, precipitated once in acidic MeOH (50 mL MeOH and 1 mL concentrated HCl) and twice in pure MeOH. Yield: 70%

^1^H NMR (400 MHz, CDCl_3_): δ 7.16 (s, 3H), 6.79 (d, J = 6.7 Hz, 4H), 5.83 (s, 2H), 4.03 (t, J = 5.8 Hz, 4H), 3.46 (q, J = 6.4 Hz, 4H), 2.01 (m, 4H), 1.97 (s, 6H), 1.41 (s, 547H), 1.10 (s, 1762H).

To solution of PIB-NH_2_ (100 mg, 0.00645 mmol) in THF (5 mL) was added DIPEA (3 eq.), PyBOP (1.25 eq.) and N-acetyl-L-alanine (1 eq.). The mixture was stirred for 24 hours. The polymer was washed twice with water, precipitated once in acidic MeOH (50 mL MeOH and 1 mL concentrated HCl) and twice in pure MeOH. Yield: 85%

^1^H NMR (400 MHz, CDCl_3_): δ 7.16 (s, 3H), 6.80 (d, J = 8.9 Hz, 4H), 6.35 (t, 2H), 6.09 (d, J = 7.0 Hz, 2H), 4.42 (p, J = 6.9 Hz, 2H), 4.02 (t, J = 5.8 Hz, 4H), 3.47 (q, J = 6.0 Hz, 4H), 2.02 (m, 4H), 1.98 (s, 6H), 1.41 (s, 535H), 1.11 (s, 1774H).

To solution of PIB-NH_2_ (100 mg, 0.00645 mmol) in THF (5 mL) was added DIPEA (3 eq.), PyBOP (1.25 eq.) and Fmoc-Ala-Ala-OH (1 eq.). The mixture was stirred for 24 hours, after which it was diluted with hexane (50 mL). The solution was then washed once with 1 M HCl, twice with water, and once with brine. The polymer was precipitated in MeOH twice. Yield: 96%

^1^H NMR (400 MHz, CDCl_3_): δ 7.76 (d, J = 7.4 Hz, 4H), 7.57 (d, J = 7.4 Hz, 4H), 7.40 (t, J = 7.1 Hz, 4H), 7.31 (t, J = 7.4 Hz, 4H), 7.16 (s, 3H), 6.79 (d, J = 8.8 Hz, 4H), 6.48 (d, J = 7.4 Hz, 2H), 6.41 (s, 2H), 5.22 (s, 2H), 4.42 (m, 6H,), 4.20 (m, Hz, 4H), 4.00 (t, J = 6.0 Hz, 4H), 3.47 (p, J = 5.5 Hz, 4H), 1.98 (m, 4H), 1.41 (s, 547H), 1.11 (s, 1742H).

PIB-(Ala)_2_-Fmoc (370 mg, 0.0242 mmol) was dissolved in 20 mL of CHCl_3_. Then, 2.1 mL (21 mmol) of piperidine was added to the solution. The mixture was stirred for of 72 hours. Subsequently, the solution was concentrated and precipitated five times in MeOH. Yield: 83%

^1^H NMR (400 MHz, CDCl_3_): δ 7.69 (d, J = 7.6 Hz, 2H), 7.16 (s, 3H), 6.80 (d, J = 8.8 Hz, 4H), 6.53 (t, 1H), 4.39 (d, J = 14.8 Hz, 2H), 4.00 (d, J = 11.7 Hz, 4H), 3.5 (m, 2H), 3.42 (p, J = 7.1 Hz, 4H), 1.99 (p, J = 6.2 Hz, 4H), 1.41 (s, 557H), 1.11 (s, 1753H).

PIB-(Ala)_2_-NH_2_ (140 mg, 0.00894 mmol) was dissolved in THF (10 mL) and acetyl chloride (2 eq.) and pyridine (2.5 eq.) were added. The resultant mixture was stirred for a period of 48 hours. The polymer was precipitated on thrice in MeOH and once in 1 M HCl. Yield: 80%

^1^H NMR (400 MHz, CDCl_3_): δ 7.16 (s, 3H), 6.80 (d, J = 8.9 Hz, 4H), 6.62 (d, J = 6.6 Hz, 2H), 6.39 (t, 2H), 6.03 (d, J = 6.0 Hz, 2H), 4.39 (p, J = 21.2, 7.1 Hz, 4H), 4.01 (t, J = 5.8 Hz, 4H), 3.47 (q, J = 6.2 Hz, 4H), 2.01 (s, 6H), 1.98 (p, J = 5.8 Hz, 4H), 1.41 (s, 530H), 1.11 (s, 1732H).

To solution of PIB-Ala_2_-NH_2_ (100 mg, 0.00645 mmol) in THF (5 mL) was added DIPEA (3 eq.), PyBOP (1.25 eq.) and N-acetyl-L-alanine (1 eq.). The mixture was stirred for 24 hours. The polymer was washed twice with water, precipitated once in acidic MeOH (50 mL MeOH and 1 mL concentrated HCl) and twice in pure MeOH. Yield: 80%

^1^H NMR (400 MHz, CDCl_3_): δ 6.81 (m, 2H), 6.60 (d, J = 7.3 Hz, 1H), 6.38 (m, 1H), 6.01 (d, J = 7.2 Hz, 1H), 4.40 (dq, J = 14.2, 7.0 Hz, 2H), 4.01 (t, J = 5.8 Hz, 2H), 3.46 (p, J = 6.0 Hz, 2H), 2.01 (s, 4H), 1.80 (s, 2H), 1.41 (s, 527H), 1.11 (s, 1562H).


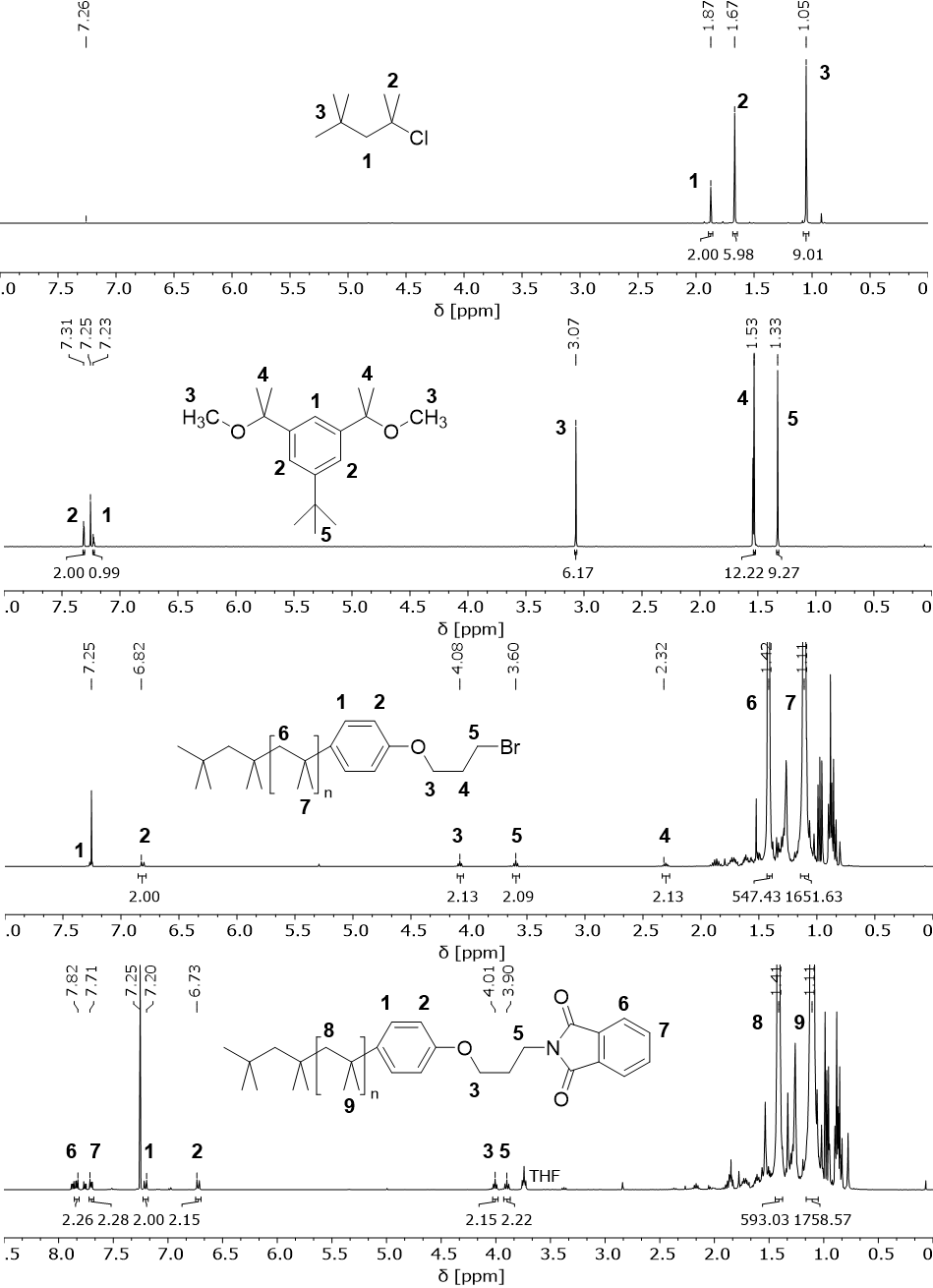


**Figure S1.** ^1^H-NMR of TMPCL, 1-(tert-butyl)-3,5-bis(2-methoxypropan-2-yl)benzene, PIB-Br and PIB-phthalimide measured in CDCl_3_ at 27°C.


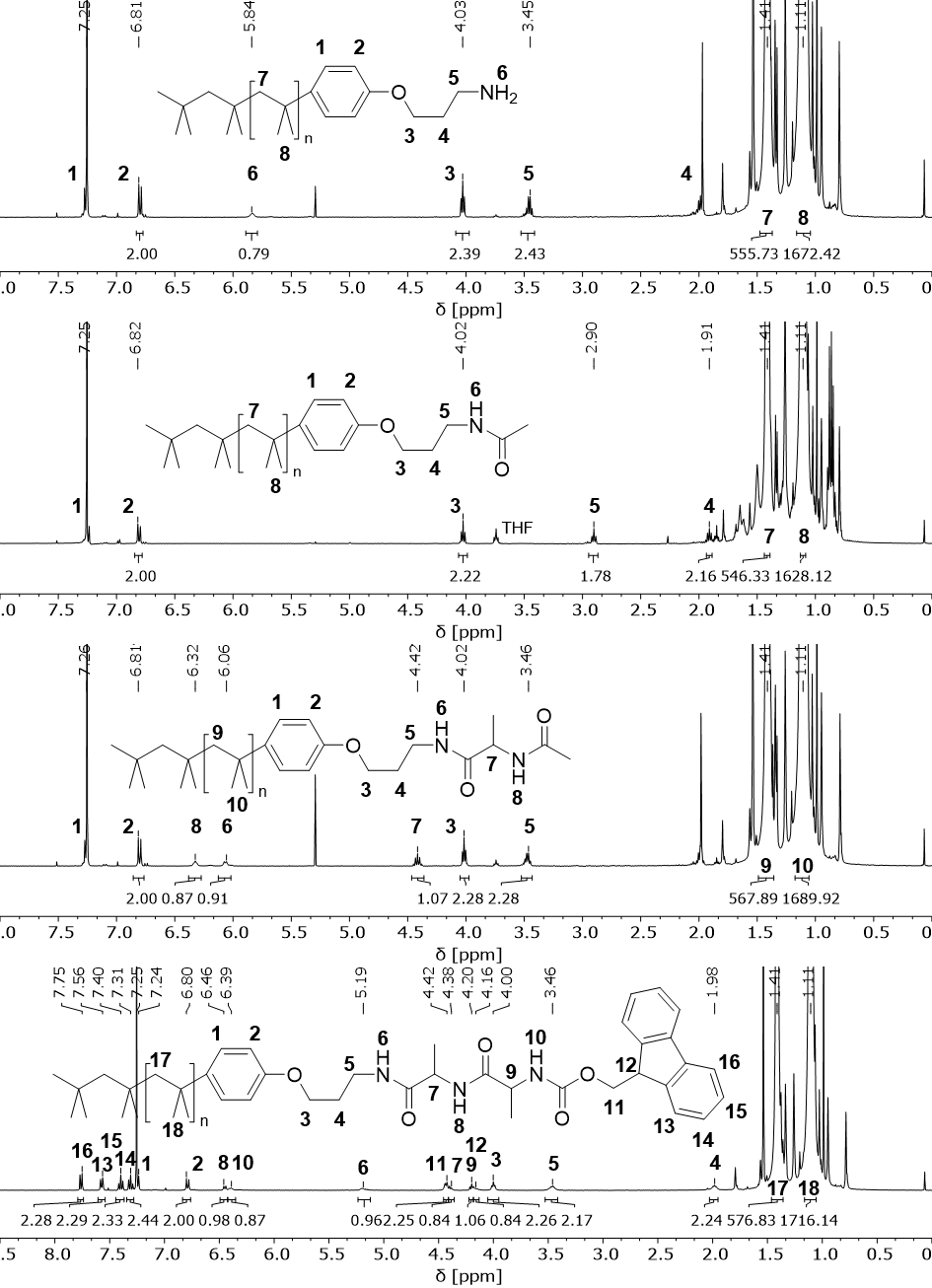


**Figure S2.** ^1^H-NMR of PIB-NH_2_, PIB-Ac **(1)**, PIB-Ala-Ac **(2)** and PIB-Ala_2_-Fmoc measured in CDCl_3_ at 27°C.


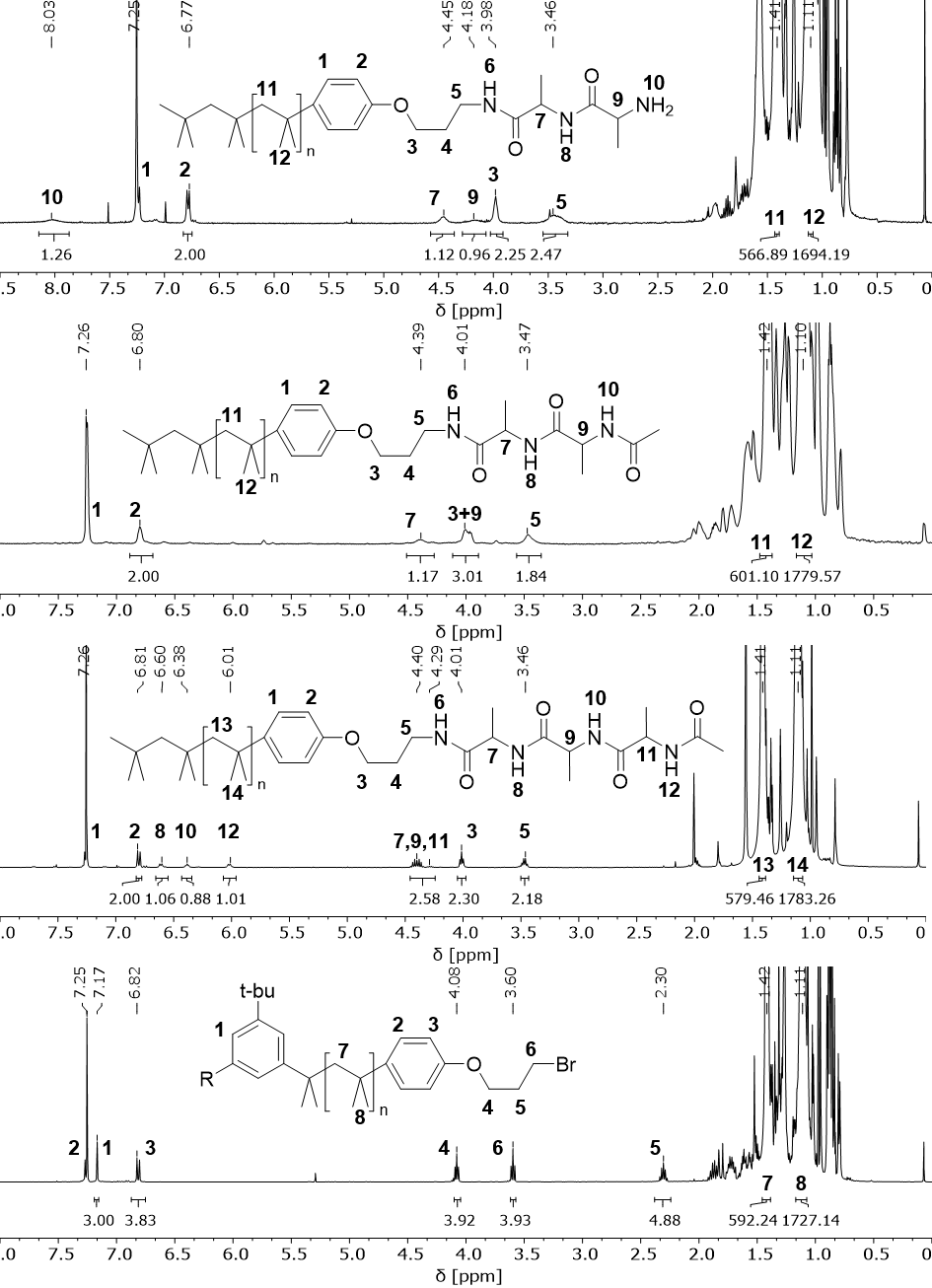


**Figure S3.** ^1^H-NMR of PIB-Ala_2_-NH_2_, PIB-Ala_2_-Ac **(3)** and PIB-Ala_3_-Ac **(4)** and PIB-Br_2_ measured in CDCl_3_ at 27°C.


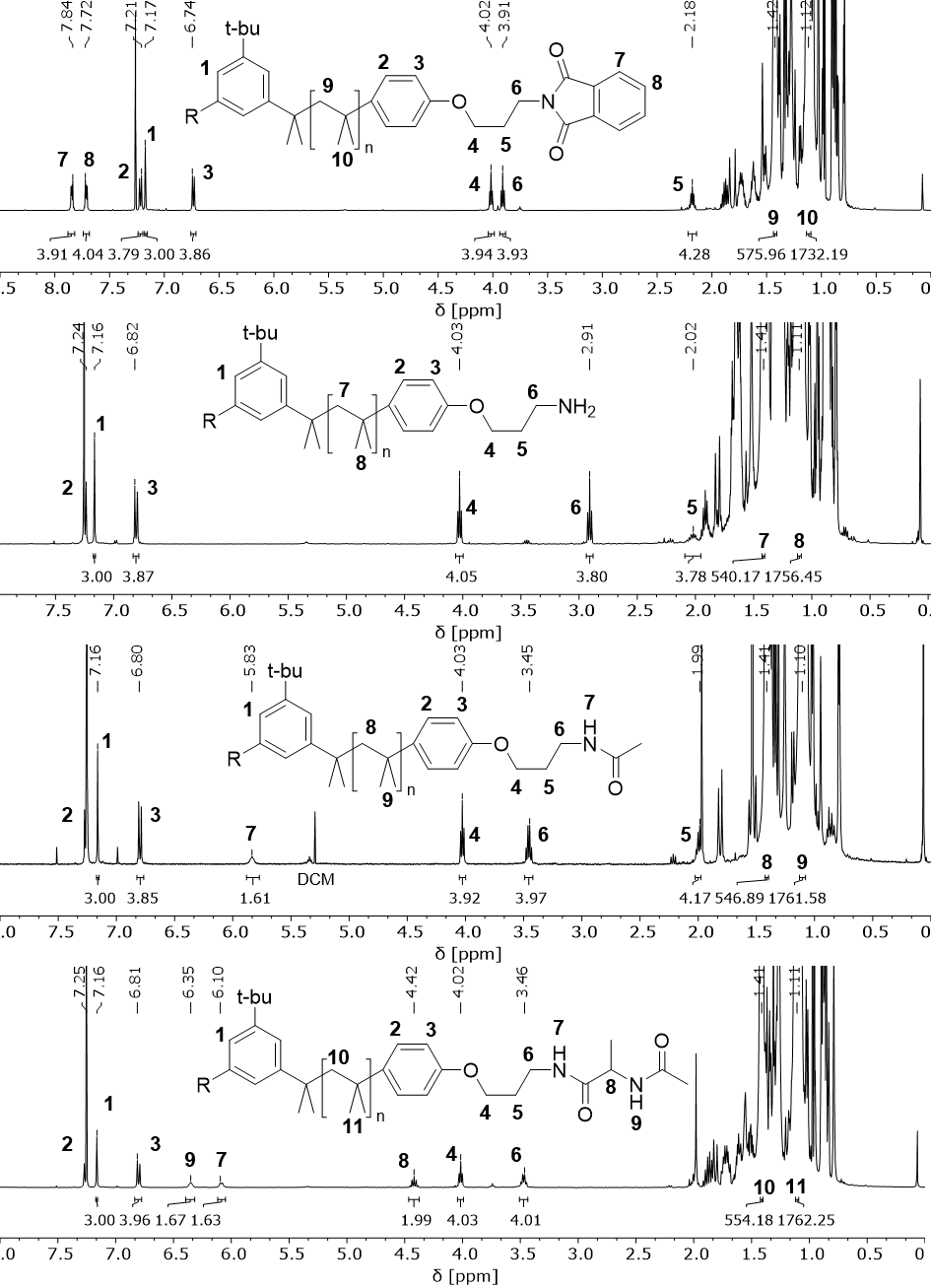


**Figure S4.** ^1^H-NMR of PIB-phthalimide_2_, PIB-(NH_2_)_2_, PIB-(Ac)_2_ **(5)** and PIB-(Ala-Ac)_2_ **(6)** measured in CDCl_3_ at 27°C.


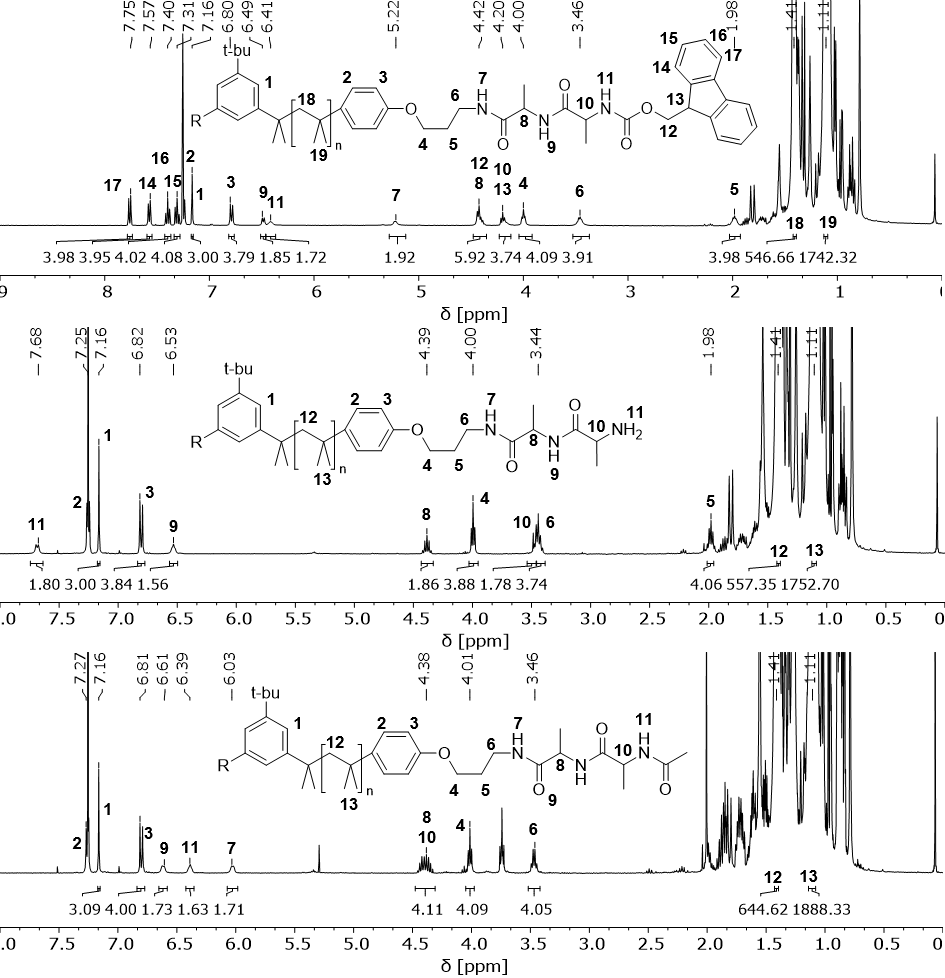


**Figure S5.** ^1^H-NMR of PIB-(Ala_2_-Fmoc)_2_, PIB-(Ala_2_-NH_2_)_2_ and PIB-(Ala_2_-Ac)_2_ **(7)** measured in CDCl_3_ at 27°C.

**Figure S6.** GPC measurements of the starting polymers as well as final polymers **(1)**, **(2)**, **(3)**, **(4)**, **(5)**, **(6)** and **(7)**

Material and Methods

If not mentioned otherwise, the chemicals were purchased from Sigma-Aldrich. Triamcinolone acetonide (9α-fluor-11β,16α,17α,21-tetrahydroxy-1,4-pregnadien-3,20-dion-16,17-acetonid) was purchased from ABCR. Nimodipine (3-(2-Methoxyethyl) 5-propan-2-yl 2,6-dimethyl-4-(3-nitrophenyl)-1,4-dihydropyridine-3,5-dicarboxylate) was bought from TCI. Sodium chloride was obtained from Roth. Na_2_HSO_4_ was purchased from Alfa Aesar. KH_2_SO_4_ was purchased from Roanal. Fmoc-Ala-Ala-OH, ((S)-2-((S)-2-((((9H-Fluoren-9-yl) methoxy) carbonyl) amino) ropanamido) propanoic acid) was bought from carbolution. All substances used were of analytical grade or higher and used without further purification if not stated otherwise. Double-distilled water was used in the experiments.

The ^1^H-NMR and ^13^C-NMR spectra were conducted on a Varian FT-NMR spectrometer (400/500 MHz and 100/125 MHz, respectively, Agilent Technologies, Waldbronn, Germany). The Measuring temperature was set to 27°C. Deuterated chloroform (CDCl_3_) was used as a solvent. All NMR spectral analysis was done using MestReNova (Version: 12.0.2-20910, Mestrelab Research, Santiago de Compostela, Spain).

The synthesized polymers were analyzed via gel permeation chromatography (GPC) using a Viscothek GPCmax VE2001 (ViscoTec, Tönning a. Inn, Germany), equipped with two columns (CLM-3008, CLM-3011) and a refractive index (RI) detector (Viscothek 3580). Linear poly-isobutylene (PIB) was used for standard calibration. THF (HPLC grade, Prolabo) was used as an elution solvent at a flow rate of 1 mL/min. The injection concentration was between 4 mg/mL and 5 mg/mL.

**Table S1.** List of all synthesized polymers.

| sample | type | M_n_(GPC) [Da] | M_n_(NMR) [Da] | PDI |
| --- | --- | --- | --- | --- |
| PIB-Br | mono | 12700 | 15400 | 1.39 |
| PIB-phthalimide | mono | --- | 15800 | --- |
| PIB-NH_2_ | mono | 13100 | 15300 | 1.31 |
| PIB-Ac | mono | 12300 | 15500 | 1.39 |
| PIB-Ala-Ac | mono | 12100 | 15900 | 1.48 |
| PIB-Ala_2_-Fmoc | mono | --- | 16100 | --- |
| PIB-Ala_2_-NH_2_ | mono | --- | 15800 | --- |
| PIB-Ala_2_-Ac | mono | 12400 | 15400 | 1.41 |
| PIB-Ala_3_-Ac | mono | 12800 | 16000 | 1.38 |
| PIB-(Br)_2_ | bi | 15500 | 15900 | 1.21 |
| PIB-(phthalimide)_2_ | bi | 15400 | 16000 | 1.25 |
| PIB-(NH_2_)_2_ | bi | 14900 | 16300 | 1.31 |
| PIB-(Ac)_2_ | bi | 15500 | 16300 | 1.25 |
| PIB-(Ala-Ac)_2_ | bi | 15500 | 16500 | 1.25 |
| PIB-(Ala_2_-Fmoc)_2_ | bi | 15500 | 16700 | 1.21 |
| PIB-(Ala_2_-NH2)_2_ | bi | 15400 | 16700 | 1.25 |
| PIB-(Ala_2_-Ac)_2_ | bi | 16700 | 16600 | 1.21 |

Fourier-transform infrared spectroscopy measurements were done on a Bruker Tensor VERTEX 70 spectrometer and Opus 8.2 was used for data analyzing. For Attenuated total reflection-infrared (ATR-FTIR), a Golden Gate Heated Diamond ATR Top-plate with temperature control was equipped.

**Figure S7.** IR-spectra of **(1)**, **(2)**, **(3)**, **(5)** and **(6)** for the relevant regions of 3200 cm^-1^ and 1600‑1700 cm^-1^.

Differential scanning calorimetry (DSC) (Netzsch DSC 204 F1 Phoenix, Netzsch, Selb, Germany) was used to analyze the thermal properties and phase transitions of the synthesized polymers. The sample (10 mg) was heated from -100°C to 200°C, using a heating rate of 10 K/min and a nitrogen atmosphere. The data were collected in the second heating cycle. Nitrogen (20 mL/min) was used as an inert gas. Data analysis was performed on the software NETZSCH Proteus (version 5.2.1, Netzsch, Selb, Germany) and Origin 2019 (OriginLab Corporation, Northampton, MA, USA).

**Figure S8.** DSC graphs of several polymers in the second heating cycle.

CD Spectroscopy was performed on a JASCO J-1500 with a DRCD-575 Ulbricht sphere at a wavelength range from 180 to 400 nm with a scanning speed of 100 nm/min with 5 accumulations. Baseline subtraction has been conducted to obtain the corrected spectra. The samples were solution cast from THF onto a glass slide, dried at room temperature at reduced pressure and measured in transmission. Data analysis was performed by Spectra Analysis and the secondary structure content was estimated by CD multivariate SSE program of JASCO Spectra Manager^TM^.

**Figure S9.** Solid state CD-spectra of **(5)**, **(6)** and **(7)** in comparison with unmodified PIB (top left), mono- and bivalent polymers, able to form beta-sheets in solid state, and a 50:50 mixture of PIB-Ala_2_-Ac **(3)** and PIB-(Ala_2_-Ac)_2_ **(7)** (top right), as well as the changes after drug incorporation of TA at 5 wt% (bottom).

Rheology experiments were performed with an MCR 101-DSO (Anton Paar, Graz, Austria) using a parallel plate geometry (diameter 8 mm). The measurements were performed from between 20°C and 200°C with 20 min of equilibration time in between the measurements. The shear rate was set in the range of 0.1 ‑ 100 s^‑1^. Data analysis was performed with the software RheoCompassTM (version V1.30.1064, Anton Paar, Graz, Austria) and Origin 2019 (OriginLab Corporation, Northampton, MA, USA).

**
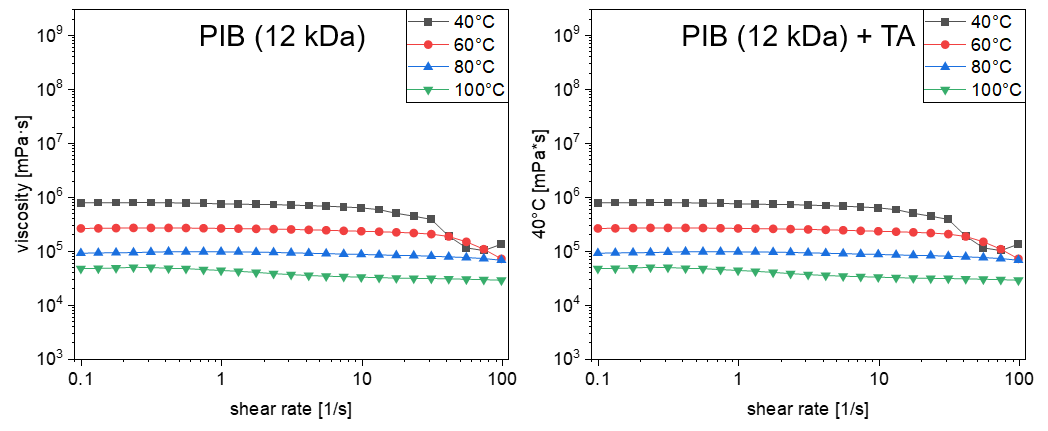
**

**Figure S10.** Rheological measurements of unmodified virgin PIB (left) as well as unmodified PIB with incorporated drug (TA, 5 wt%) (right).

Grazing incidence wide angle x-ray scattering (GIWAXS) was performed at room temperature using a Retro-F laboratory setup (SAXSLAB, Copenhagen, Denmark) equipped with a microfocus X-ray source (AXO Dresden GmbH, Dresden, Germany) and an ASTIX multilayer X-ray optics (AXO Dresden GmbH) as monochromator, resulting in CuKα radiation with a wavelength of 0.154 nm. The diameter of the circular beam was about 1.5 mm and the exposure time was 7200 s. The scattered X-ray intensity was recorded by a two-dimensional PILATUS3 R 300K detector (DECTRIS Ltd., Baden, Switzerland), with the sample-to-detector distance of about 550 mm. Thin films were prepared on a stainless steel plate, cast from solution and dried in vacuum for at least 24 h and measured at an angle of 0.2°.

**Figure S11.** GIWAXS measurements of a 50:50 mixture of PIB-Ala_2_-Ac and PIB-(Ala_2_-Ac)_2_ mixture compared with the same composition but with TA incorporation (5 wt%) (left: full range, right: magnified in the relevant range of the PIB-halo).

AFM measurements were performed using a nanosurf CoreAFM with Tap190AI-G Cantilevers in the Phase-contrast mode. Data analysis was done using Gwyddion 2.53 (freeware, http://gwyddion.net/).


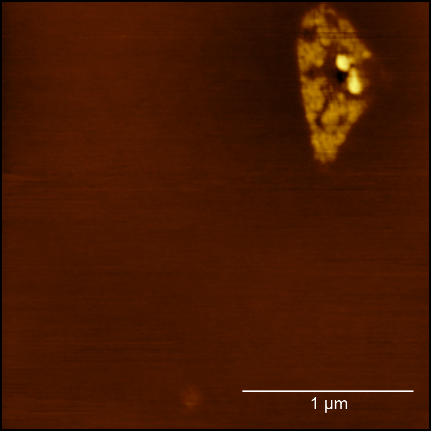

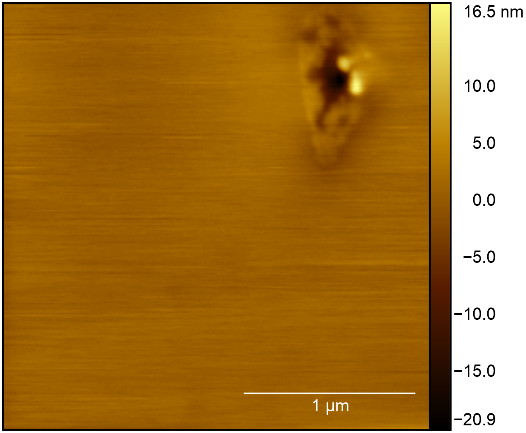


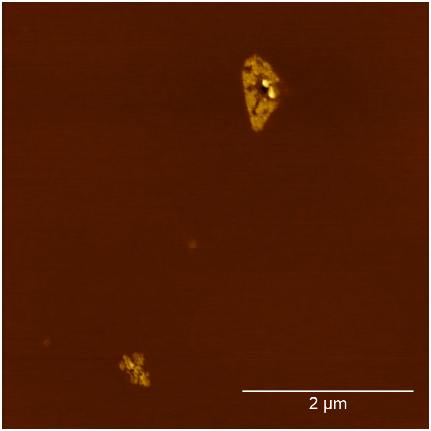

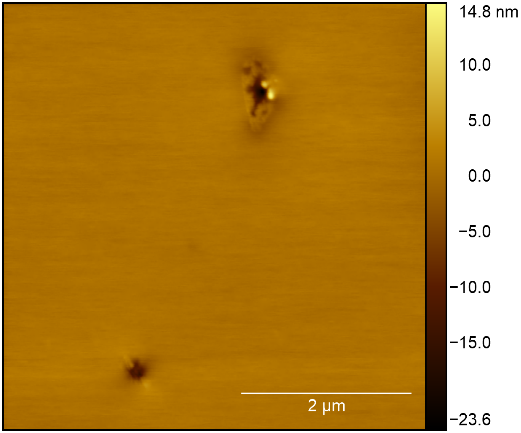


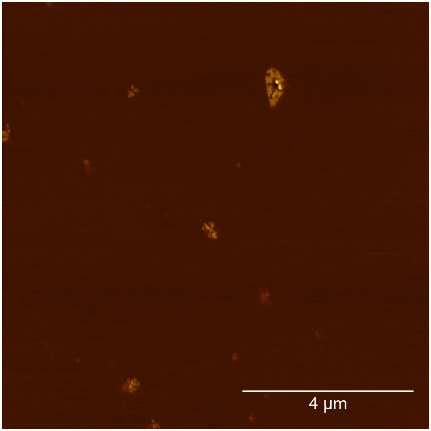

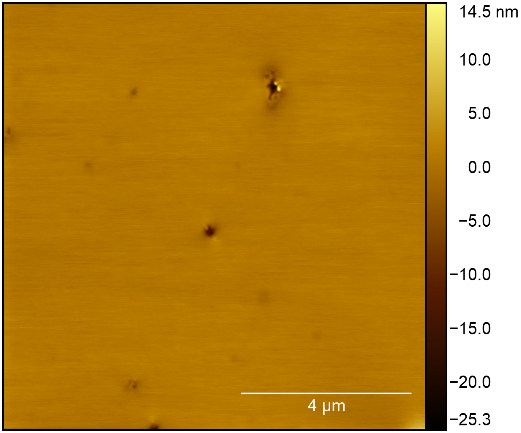


**Figure S12.** phase images (left) and height images (right) of PIB-Ala_2_-Ac with TA incorporation (5 wt%) taken with AFM at different magnifications (top: 2.5 µm, middle: 5 µm, bottom: 10 µm).


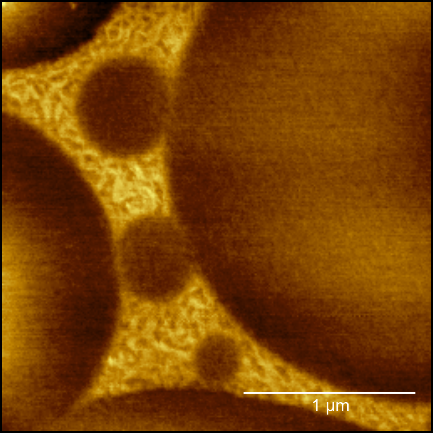

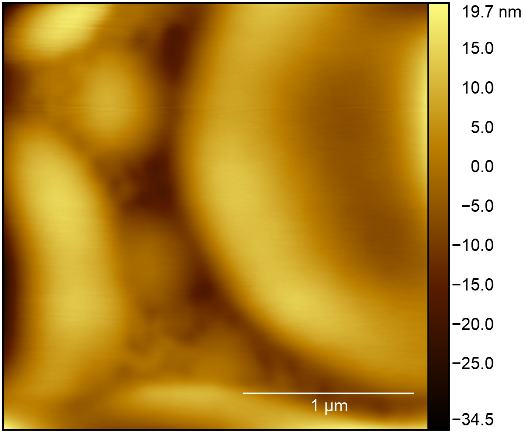


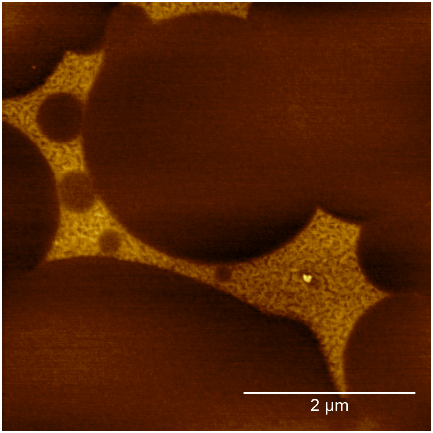

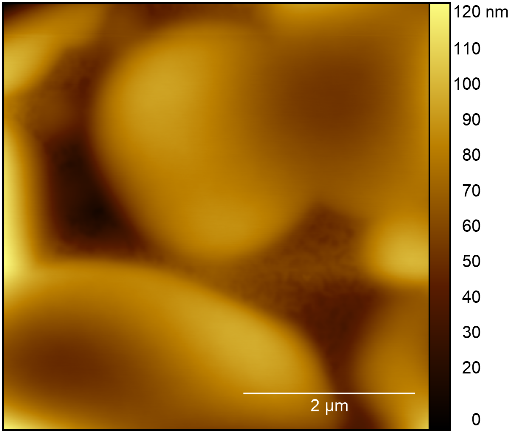


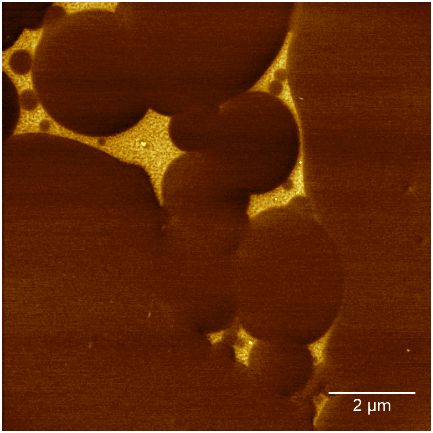

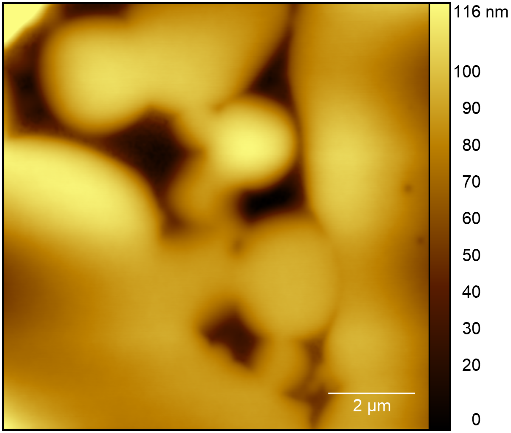


**Figure S13.** phase images (left) and height images (right) of PIB-(Ala_2_-Ac)_2_ with TA incorporation (5 wt%) taken with AFM at different magnifications (top: 2.5 µm, middle: 5 µm, bottom: 10 µm).


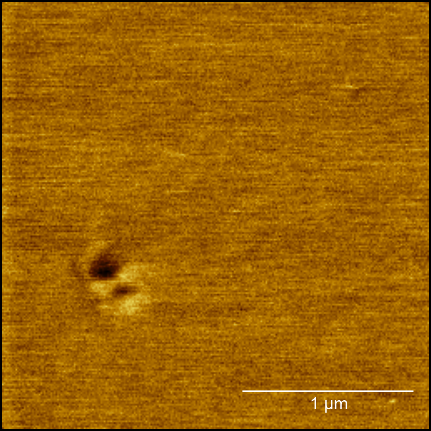

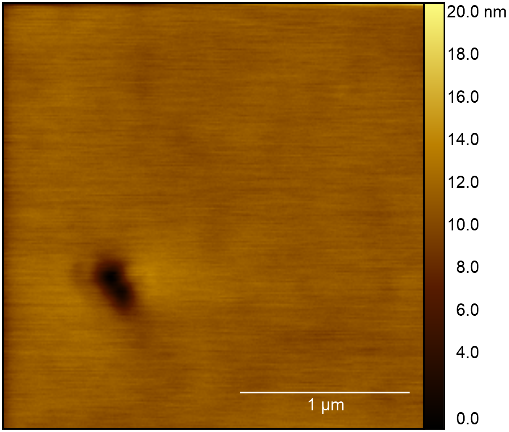


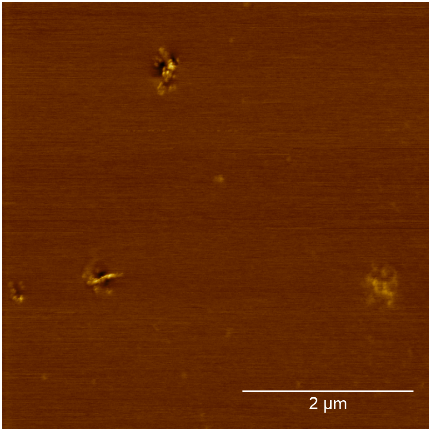

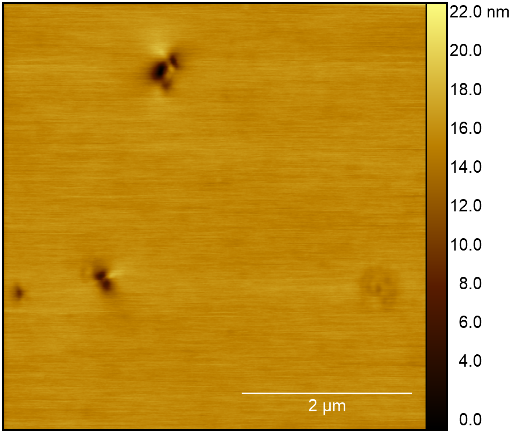


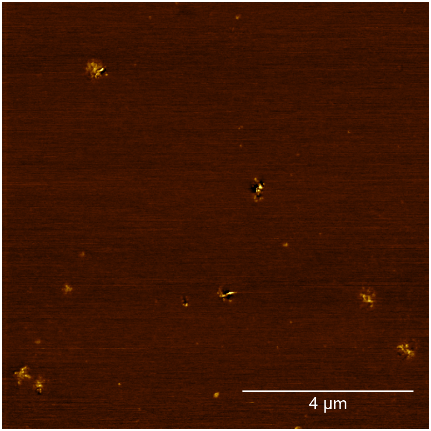

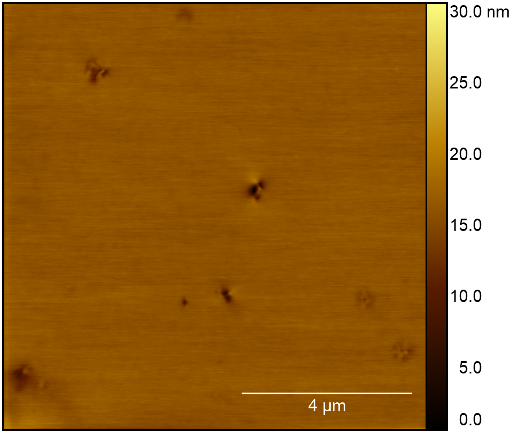


**Figure S14.** phase images (left) and height images (right) of PIB-Ala_2_-Ac with NMP incorporation (5 wt%) taken with AFM at different magnifications (top: 2.5 µm, middle: 5 µm, bottom: 10 µm).


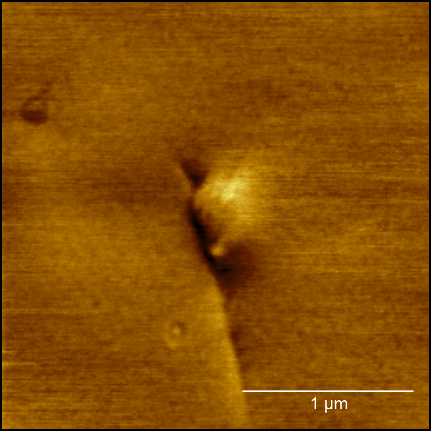

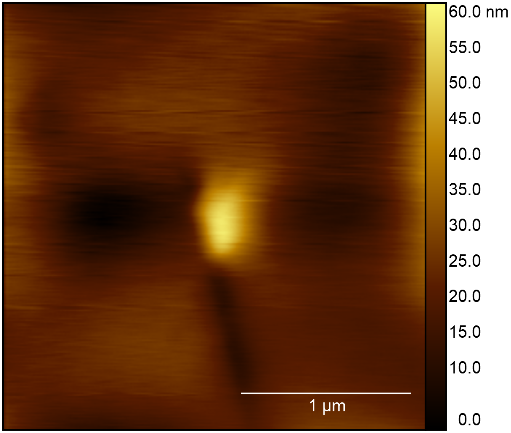


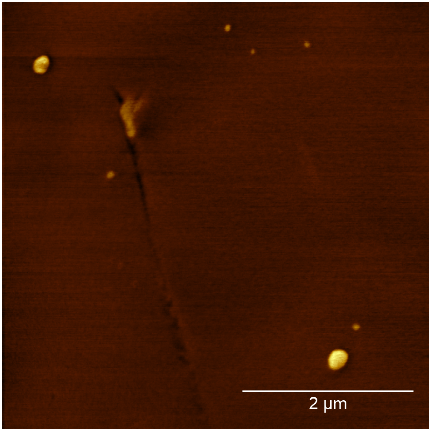

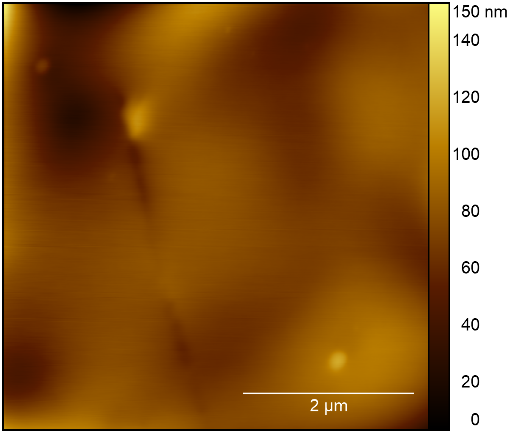


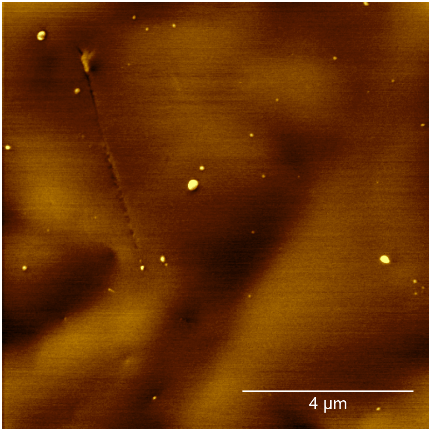

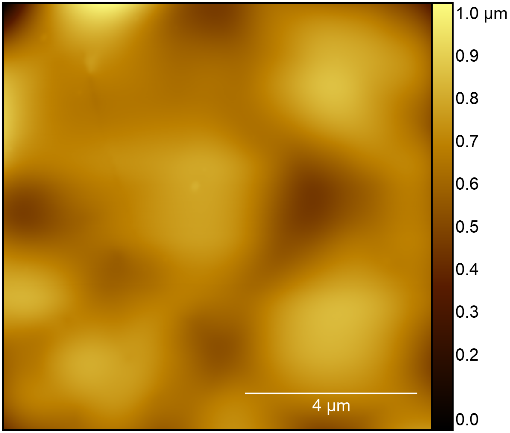


**Figure S15.** phase images (left) and height images (right) of PIB-(Ala_2_-Ac)_2_ with NMP incorporation (5 wt%) taken with AFM at different magnifications (top: 2.5 µm, middle: 5 µm, bottom: 10 µm).


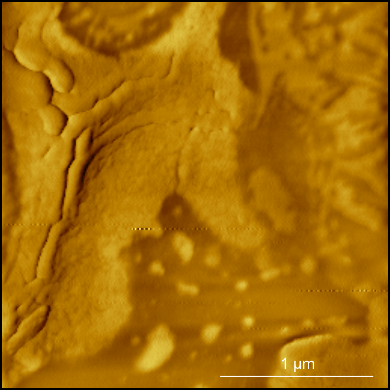

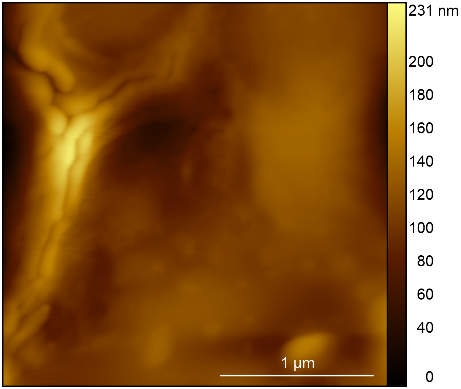


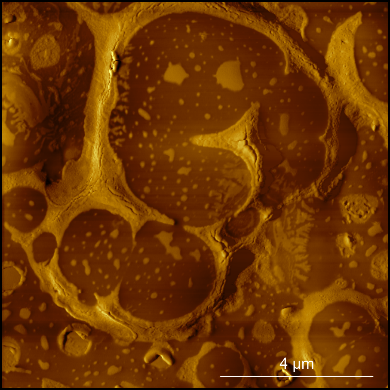

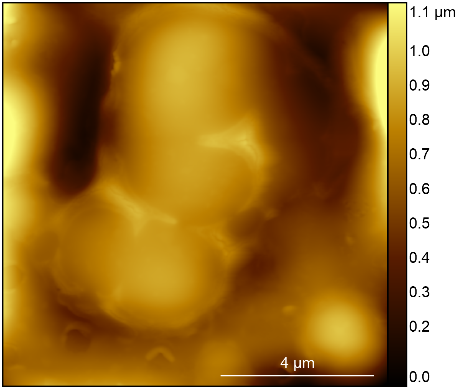


**Figure S16.** phase images (left) and height images (right) of a 50:50 mixture of PIB-Ala_2_-Ac and PIB-(Ala_2_-Ac)_2_ mixture with TA incorporation (5 wt%) taken with AFM at different magnifications (top: 2.5 µm, bottom: 10 µm).


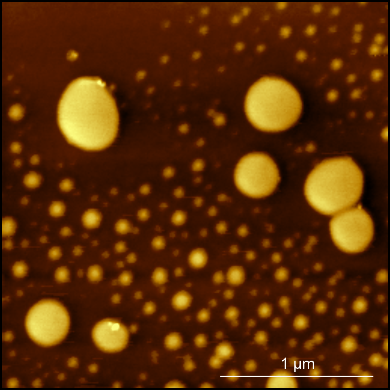

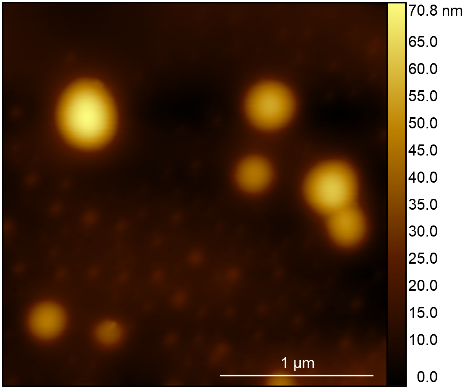


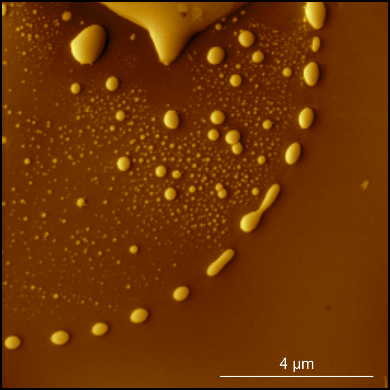

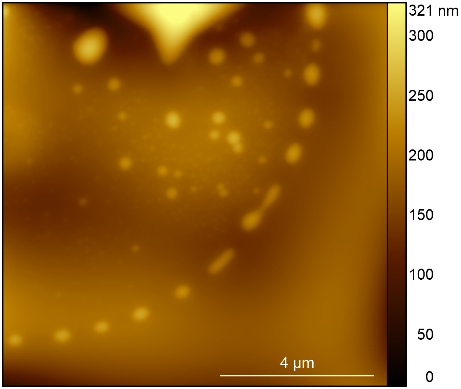


**Figure S17.** phase images (left) and height images (right) of a 50:50 mixture of PIB-Ala_2_-Ac and PIB-(Ala_2_-Ac)_2_ mixture with NMP incorporation (5 wt%) taken with AFM at different magnifications (top: 2.5 µm, bottom: 10 µm).

Drug release studies were performed in a phosphate-buffered saline (PBS), pH 7.4, at 37°C for all samples over a time period of 42 days. The shaking speed was set to 150 min^-1^. After each time step (6 h, 12 h, 24 h, 48 h, 4 d, 7 d, 14 d, 28 d, 42 d) the solvent was completely exchanged, to ensure sink conditions.

For quantification of triamcinolone acetonide, high-performance liquid chromatography (HPLC) was used. Experiments were conducted on a Nova-Pak C18 4 µm (4.6 x 250 mm) column, which needed to be replaced during the experiment, switching to an Atlantis T3 5 µm (4.6 x 250 mm) column. A mixture of ACN, water, and formic acid (60:40:0.1) was used as a solvent. Solutions of the two drugs in methanol in a range of 0.001-0.2 mg/mL were used to obtain a calibration curve **(Figure S17, S18)**. Due to the column change, two extra calibrations needed to be done. To determine the total drug load, samples were dissolved in 0.2 mL THF. Methanol (0.8 µL) was added to precipitate the polymer and the sample was then filtered using a 0.2 µm PTFE filter to remove the precipitate.

**Figure S18.** Linear fits for calibration using the Nova-Pak C18 column (left) and the Atlantis T3 (right) for nimodipine.

**Figure S19.** Linear fits for calibration using the Nova-Pak C18 column (left) and the Atlantis T3 (right) for triamcinolone acetonide.
